# Pregnant Women’s Views on the Feasibility and Acceptability of Web-Based Mental Health E-Screening Versus Paper-Based Screening: A Randomized Controlled Trial

**DOI:** 10.2196/jmir.6866

**Published:** 2017-04-07

**Authors:** Dawn Kingston, Marie-Paule Austin, Sander Veldhuyzen van Zanten, Paula Harvalik, Rebecca Giallo, Sarah D McDonald, Glenda MacQueen, Lydia Vermeyden, Gerri Lasiuk, Wendy Sword, Anne Biringer

**Affiliations:** ^1^ Faculty of Nursing University of Calgary Calgary, AB Canada; ^2^ University of New South Wales Sydney Australia; ^3^ Division of Gastroenterology University of Alberta Edmonton, AB Canada; ^4^ Murdoch Childrens Research Institute, Royal Children's Hospital Parkville Australia; ^5^ McMaster University Hamilton, ON Canada; ^6^ University of Calgary Calgary, AB Canada; ^7^ College of Nursing University of Saskatchewan Regina, SK Canada; ^8^ University of Ottawa Ottawa, ON Canada; ^9^ Department of Family and Community Medicine University of Toronto Toronto, ON Canada

**Keywords:** pregnancy, mental health, screening, prenatal care, computers

## Abstract

**Background:**

Major international guidelines recommend mental health screening during the perinatal period. However, substantial barriers to screening have been reported by pregnant and postpartum women and perinatal care providers. E-screening offers benefits that may address implementation challenges.

**Objective:**

The primary objective of this randomized controlled trial was to evaluate the feasibility and acceptability of Web-based mental health e-screening compared with paper-based screening among pregnant women. A secondary objective was to identify factors associated with women’s preferences for e-screening and disclosure of mental health concerns.

**Methods:**

Pregnant women recruited from community and hospital-based antenatal clinics and hospital-based prenatal classes were computer-randomized to a fully automated Web-based e-screening intervention group or a paper-based control group. Women were eligible if they spoke or read English, were willing to be randomized to e-screening, and were willing to participate in a follow-up diagnostic interview. The intervention group completed the Antenatal Psychosocial Health Assessment and the Edinburgh Postnatal Depression Scale on a tablet computer, while controls completed them on paper. All women completed self-report baseline questions and were telephoned 1 week after randomization by a blinded research assistant for a MINI International Neuropsychiatric Interview. Renker and Tonkin’s tool of feasibility and acceptability of computerized screening was used to assess the feasibility and acceptability of e-screening compared with paper-based screening. Intention-to-treat analysis was used. To identify factors associated with preference for e-screening and disclosure, variables associated with each outcome at P<.20 were simultaneously entered into final multivariable models to estimate adjusted odds ratios (AORs) and 95% CIs.

**Results:**

Of the 675 eligible women approached, 636 agreed to participate (participation rate 94.2%) and were randomized to the intervention (n=305) or control (n=331) groups. There were no significant baseline differences between groups. More women in the e-screening group strongly or somewhat agreed that they would like to use a tablet for answering questions on emotional health (57.9%, 175/302 vs 37.2%, 121/325) and would prefer using a tablet to paper (46.0%, 139/302 vs 29.2%, 95/325), compared with women in the paper-based screening group. There were no differences between groups in women’s disclosure of emotional health concerns (94.1%, 284/302 vs 90.2%, 293/325). Women in the e-screening group consistently reported the features of e-screening more favorably than controls (more private or confidential, less impersonal, less time-consuming). In the multivariable models, being in the e-screening group was significantly associated with preferring e-screening (AOR 2.29, 95% CI 1.66-3.17), while no factors were significantly associated with disclosure.

**Conclusions:**

The findings suggest that mental health e-screening is feasible and acceptable to pregnant women.

**Trial Registration:**

Clinicaltrials.gov NCT01899534; https://clinicaltrials.gov/ct2/show/NCT01899534 (Archived by WebCite at http://www.webcitation.org/6ntWg1yWb)

## Introduction

### The Need for Mental Health Screening

Depression and anxiety are among the most common morbidities in pregnancy, with prevalence rates of 13%-29% [1-3], and are the leading causes of maternal mortality in Western countries [4]. Without early screening and treatment, 50% to 70% of women with prenatal anxiety or depression symptoms [5] will experience persistent symptoms through their child’s early years [6,7]. Recent findings from longitudinal birth cohorts also reveal that chronicity of depression starting in pregnancy, whether severe or subclinical, is emerging as a major risk factor in child development and mental health [8-11].

In the absence of routine screening, mental health problems are severely underdetected and undertreated in perinatal settings [12,13]. This is, in part, due to the fact that the majority of pregnant and postpartum women do not volunteer information about their mental health without being prompted by a perinatal care provider [14-18]. In our previous studies, we reported that while 67% of pregnant women did not raise concerns about their mental health with their physician or obstetrician, nurse, or midwife, 97% indicated they were comfortable with provider-initiated screening [19]. Our studies have identified other common barriers that deter women from self-identifying mental health problems, including “false” reassurance that they have received from friends or family, not knowing whether their symptoms are “normal” or not during pregnancy, and stigma-induced concerns such as not wanting their care provider to see them as depressed or anxious and not wanting to be seen as a bad mother [19]. However, 79% of pregnant women reported they would disclose mental health concerns if asked as part of their routine prenatal care [19,20]. Clearly, routine, provider-initiated mental health screening plays an important role in women’s willingness to disclose prenatal mental health problems.

Studies also show that underdetection of mental health problems in the perinatal population is due to the lack of standardized screening, where up to 80% of cases remain unidentified by perinatal care providers who used unstandardized approaches for detection of mental health disorders (eg, without a validated tool) [21,22]. However, recent studies reveal that routine prenatal and postnatal screening enhances detection and increases the likelihood that women with a positive screening result for depression will link to mental health services [23,24]. Thus, antenatal mental health screening holds benefits for enhanced detection, increased linkage to services, and improved clinical outcomes.

Although current evidence and international guidelines from the United Kingdom [25], Australia [26], and the United States [27,28] support the need for antenatal screening as a key intervention for interrupting the cycle of perinatal mental disorders and their negative impact on maternal and child well-being, serious challenges exist for its implementation. Despite high acceptance by women [29-31] and providers [32-35], only 20% of North American perinatal care providers conduct proactive screening as part of prenatal care [36] and less than 15% of pregnant or postpartum women receive the help they need [37]. A systematic review conducted by our team (manuscript in preparation) found that substantial personal and system-based barriers to routine screening exist for health care providers, including lack of time to screen, lack of accurate assessment tools and knowing how to interpret them, lack of defined referral processes, the absence of connections with mental health services, and frustration with the lack of availability of timely services for their pregnant patients [38-40].

Taken together, this body of research underscores the need to develop screening processes that are feasible for and acceptable to both women and service providers, are designed to overcome barriers to implementation, and are cost-effective and clinically useful. Indeed, the most effective perinatal mental health screening and management programs are those characterized by screening processes that are incorporated into routine care with designated systems of referral and treatment that are initiated immediately after screening [26,41,42].

### The Potential Impact of E-Screening

E-screening has potential to be an effective, low-resource screening approach that can be feasibly embedded in a variety of perinatal settings [43,44]. Importantly, it has potential to overcome the personal and system-based barriers to screening identified by pregnant women and health care providers, and it can screen for sensitive issues such as intimate partner violence [45-47] and postpartum depression [48]. It can increase efficiency of mental health care by reallocating scarce human resources to where they are most needed—in-depth follow-up assessment, referral, and treatment. It is well-suited for busy clinical settings, can be personalized to patient needs, offers audio or video options for low literacy, provides real-time data [43,44], achieves similar or greater rates of disclosure compared with interviews, and is preferred by patients because of its anonymity [44,45,49,50]. However, to date no studies have evaluated the feasibility or acceptability of e-screening as an approach to routine screening in pregnant women.

### Objectives

The objective of this study was to compare the views of pregnant women randomized to an intervention group and a paper-based screening control group on the feasibility (eg, disclosure of concerns about their mental health, specific features of screening) and acceptability (eg, women’s preference) of e-screening. A secondary objective was to identify factors associated with women’s preferences for e-screening and their ability to disclose mental health concerns.

## Methods

### Study Design

**Figure 1:**
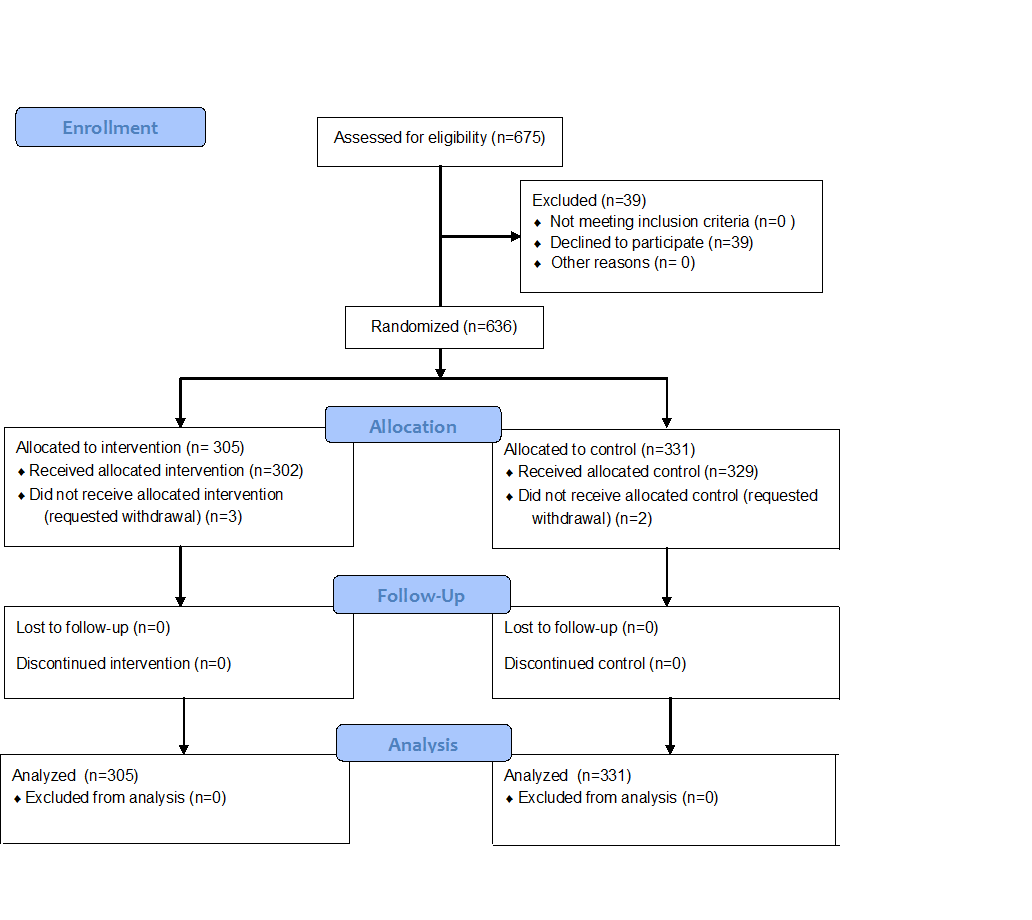
CONSORT flow diagram.

The study was a parallel-group, randomized controlled superiority trial (Figure 1). The protocol has been previously published (ClinicalTrials.gov identifier: NCT01899534) [51]. Approval for this study was granted by the Human Research Ethics Board at the University of Alberta.

### Eligibility Criteria

Pregnant women were eligible if they were (1) able to speak or read English; (2) willing to be randomized to e-screening; and (3) willing to participate in a follow-up diagnostic interview within 1 week of recruitment. Because the Web-based screening tool was intended to be completed unassisted, it was designed for use by women with varying degrees of computer literacy.

### Setting and Recruitment

Women were recruited from 2 community-based family physician–led maternity clinics, a high-risk antenatal unit in a tertiary care center, and 2 community hospital-based prenatal classes in Edmonton, Alberta. The high-risk antenatal unit is located in the inner city and serves a demographically diverse population. One maternity clinic was located in a middle-class suburb, while the other was located in a neighborhood with a high proportion of new immigrant families. The hospital-based prenatal classes served women from across the urban center. Thus, the recruitment strategy aimed to include participants with diverse demographic and obstetric characteristics. None of the care providers at the recruitment sites had specialized training in mental health care. Additional details on the recruitment sites are available in the study’s published protocol [51].

Trained research assistants at each site used a standardized script to invite women to participate in the study. Once women completed the consent form electronically on a tablet computer, the computer program designed by the Women and Children’s Health Research Institute (WCHRI) automatically randomized them (1:1) to the intervention or control group. Thus, the research assistant was blinded to group allocation. Full details on recruitment and consent procedures are found in the trial protocol [51].

### The E-Screening Intervention

The e-screening intervention was a full psychosocial assessment including (1) the Antenatal Psychosocial Health Assessment (ALPHA) [21,35] and (2) the Edinburgh Postnatal Depression Scale (EPDS) [52]. This is aligned with international guidelines recommending that screening comprise an evaluation of risks for mental health problems (ALPHA) as well as current symptoms (EPDS) [26,42].

The ALPHA asks women questions on the topics of family life, stressors, feelings about the pregnancy, substance use, abuse, and family of origin. Items are rated as yes or no or 6-point Likert-scale options and are completed in 5 minutes. On the basis of a review of responses in each category, providers subjectively judge whether women are at low, some, or high psychosocial risk. The ALPHA has undergone extensive face and content validity testing, can readily be implemented in routine prenatal care, and pregnant women find it acceptable [35,53].

The 10-item EPDS is one of the most widely used screening instruments for detecting antenatal and postnatal depression symptoms within the previous 7 days [52]. A score of 13 or higher (range 0-30) is a well-established cutoff for clinically significant depression symptoms consistent with meeting the criteria for a major depressive episode [52]. Original psychometric testing resulted in a sensitivity of 85%, specificity of 77%, positive predictive value of 83%, split-half reliability of .8, and Cronbach alpha of .87 [52]. A screenshot of the Web-based version of the EPDS is provided in Multimedia Appendix 1.

Women randomized to the intervention group accessed the intervention for free. They completed the assessment on a single occasion and had no further access to the intervention beyond that time. Research assistants recruiting the women provided technical assistance as needed to assist women to get started on the tablet computer, although the link was labeled and readily available on the home screen. No specialized training was offered to the intervention group because completion of the Web-based e-screening tool was intended to be a largely self-sufficient, self-report option for clinical settings. No study participants were aware which group was the intervention of interest.

### The Paper-Based Screening Control Group

Women in the control group completed paper-based versions of the ALPHA and EPDS, followed by the Web-based baseline questionnaire.

### Procedures

Following recruitment, women completed the Web-based e-screening or paper-based version of the screening tools independently (in the recruitment setting) as well as a Web-based baseline questionnaire including (1) demographics (age, parity, marital status, education, income, ethnicity, country of birth, and length of time in Canada); (2) obstetric history (current and past, including use of fertility treatments); (3) mental health history (diagnoses, treatment); (4) level of comfort with computer technology; (5) quality of relationship with perinatal care provider; (6) level of social support, experience of talking with doctor, nurse, or midwife about emotional health; and (7) adverse childhood experiences (using ACE questionnaire [54]). With the exception of the ACE questionnaire, questions were drawn from those used in the All Our Babies Study [55,56] and the Canadian Maternity Experiences Survey [57]. The baseline questionnaire also included Renker and Tonkin’s Computer Assessment Evaluation (CAE), which is a feasibility and acceptability assessment. Logos belonging to institutional affiliations were visible on questionnaires and consent forms, as well as in the description of the primary investigator. Women in both groups were telephoned by a trained research assistant (blinded to group allocation), 1 week after recruitment, to complete a MINI International Neuropsychiatric Interview (MINI, version 6.0.0) [58].

No data were stored on the tablets. When women submitted their information, it was sent to a secure server housed in the Faculty of Medicine & Dentistry at the University of Alberta. The Web-based e-screening tool and questionnaire were built using an existing infrastructure offered by Checkbox Survey software provided by the WCHRI Clinical Research Informatics Core. The Web-based e-screening tool was tested for usability and navigability through focus groups as part of a concurrent randomized controlled trial, as described in that trial’s published protocol [59]. No changes to the tool were made after initial testing. Data transfer between the tablet and server was encrypted. Data imported to statistical databases for analysis were not identifiable.

### Safety Protocol

If women met the criteria for a mood or anxiety disorder on the MINI or if they scored 13 or more on the EPDS, the research assistant made a referral for the women (with their permission) to the hospital-based reproductive mental health support program.

### Sample Size

Because no data were available to guide estimation of a minimal clinically important difference in true cases detected through e-screening, we used a confidence interval approach [60]. On the basis of high levels of acceptability and disclosure reported using computer screening [46,47], we established that e-screening would be feasible if, in the intervention group, 85% of women indicated that they were able to tell the truth on all questions on the psychosocial assessment (question 7 of the computer assessment evaluation, CAE). For our other outcomes, we based the sample size calculation on 85% of women scoring 4-8 on the Risk subscale of the Disclosure Expectations Scale and 85% scoring 16-20 on the Utility subscale of the Disclosure Expectations Scale. Using a margin of error of .05 and 25% as an estimated loss to follow-up, we calculated that 261 women per group (N=522) were required (see protocol [51] for calculation). At a final sample size of 636, the study is sufficiently powered to detect differences in the outcomes between groups if they exist.

### Measurement of Outcomes

We measured women’s views on the feasibility and acceptability of e-screening with an adapted 9-item version of Renker and Tonkin’s assessment of the feasibility of computerized screening for interpersonal violence [46,47]. Of the 9 items, 2 items are related to acceptability of e-screening (I did or would like to use a tablet to answer these questions about emotional health; I would prefer answering questions about emotional health on the tablet compared to a paper questionnaire) and the remaining 7 items measure broad areas of feasibility, including disclosure (I was able to tell the truth on all the questions about emotional health; I did not like answering the questions about emotional health on the tablet or paper because it was hard to answer how I really felt), features of screening (privacy; ease of understanding questions; impersonal; time to completion), and comparison with paper-based screening. Women in the intervention group answered questions rating their experience of e-screening (eg, I *liked* answering questions about emotional health on a tablet because it felt private). Those in the control group answered questions to assess their views on e-screening if they had the opportunity (eg, I *would like* answering questions about emotional health on a tablet because it would feel private). Responses were rated on a 5-point Likert scale (1=strongly disagree to 5=strongly agree).

### Analysis

Intention-to-treat analysis was conducted for all research questions. We used descriptive data (frequencies and 95% CIs; means and SDs) to describe the sample. Baseline differences in groups were compared using independent *t* tests (means) and chi-square tests (%) to determine the extent to which randomization was successful. Statistical significance for all analyses and final models was set at *P*<.05. We used chi-square tests to compare proportions of women in each group responding affirmatively to questions on feasibility and acceptability.

For analyses related to factors associated with the outcomes of preference for e-screening and disclosure, we conducted bivariate analyses to identify independent factors that were significantly associated with each of the outcomes at *P*<.20, estimating unadjusted odds ratios and their 95% CIs. Those variables were entered in the 2 final multivariable models, where *P*<.05 defined factors that were significantly associated with the outcomes in the final models. All significant independent variables were entered into the multivariable models simultaneously, and each variable was controlled for by all other variables in the model. For the final models, we reported adjusted odds ratios and their 95% CIs.

## Results

### Sample Characteristics

Of the 675 eligible women who were approached between August 2013 and January 2015, a total of 636 (94.2%) women agreed to participate and were randomized to the intervention (n=305) or control (n=331) group. No women were deemed ineligible after the initial screening for eligibility. A total of 5 women withdrew from the study following group allocation, 3 in the intervention group and 2 in the control group. There were no statistically significant differences in relation to demographic variables between the 2 groups (Table 1).

The majority of pregnant women were 25 to 34 years of age, partnered (married, common-law, or living with a partner), were white, had incomes of Can $80,000 or more, had at least some postsecondary education, were pregnant with their first child, and were in their first trimester of pregnancy (80.3%, 494/615; see Table 1). More than a quarter of the participants had been diagnosed and treated for anxiety, depression, or another mental health concern before joining the study, and 18.0% (113/629) reported having 4 or more adverse childhood experiences. There were no statistically significant differences between the intervention and control groups in demographic characteristics, comfort with computers, tablets, or mobile phone previous mental health diagnoses or treatment, obstetric history, mean gestational age, or number of adverse childhood experiences. More than two-thirds of women (n=423) were recruited from community-based maternity clinics, 21.0% (131/624) were recruited from community-based prenatal classes, and 11.2% (70/624) were recruited from the high-risk antenatal unit. Missing data were 3.3% (615/636) or less for all variables, with the majority having less than 1.5% (10/636); thus, data imputation was not used.

**Table 1:** Sample characteristics (N=636).

Characteristics		Full sample (N=636^a^), n (%)	Paper-based screening group (n=331^a^), n (%)	E-screening group (n=305^a^), n (%)	*P* value^b^
**Recruitment site**					
	Community-based clinics	423 (67.8)	224 (70.0)	199 (65.5)	.47
	High-risk antenatal unit at tertiary care center	70 (11.2)	34 (10.6)	36 (11.8)	
	Prenatal classes	131 (21.0)	62 (19.4)	69 (22.7)	
**Age, years**					
	<25	88 (13.9)	50 (15.2)	38 (12.5)	.51
	25-34	459 (72.2)	233 (70.6)	226 (74.6)	
	35+	86 (13.6)	47 (14.2)	39 (12.9)	
**Income in Can $**					
	Less than $40,000	97 (15.4)	52 (15.8)	45 (14.9)	.81
	$40,000-$79,999	139 (22.0)	75 (22.8)	64 (21.2)	
	$80,000 or more	395 (62.6)	202 (61.4)	193 (63.9)	
**Education**					
	High school or less	100 (15.8)	57 (17.3)	43 (14.2)	.29
	Some postsecondary or more	531 (84.2)	272 (82.7)	259 (85.8)	
**Marital status**					
	Unpartnered (single, divorced, or separated)	27 (4.3)	14 (4.3)	13 (4.3)	.98
	Partnered (married, common-law, or living with a partner)	604 (95.7)	315 (95.7)	289 (95.7)	
**Ethnicity**					
	Not white	169 (26.8)	91 (27.7)	78 (25.8)	.60
	White	462 (73.2)	238 (72.3)	224 (74.2)	
**Born in Canada**					
	No	119 (18.9)	66 (20.1)	53 (17.5)	.42
	Yes	512 (81.1)	263 (79.9)	249 (82.5)	
**Ever diagnosed with depression, anxiety, or any other kind** **of emotional concern**					
	Yes	164 (25.9)	86 (26.1)	78 (25.7)	.91
	No	470 (74.1)	244 (73.9)	226 (74.3)	
**Ever treated for depression, anxiety, or any other kind of** **emotional concern**					
	Yes	179 (28.2)	92 (27.9)	87 (28.6)	.84
	No	455 (71.8)	238 (72.1)	217 (71.4)	
**Pregnant before**					
	First child	426 (69.3)	213 (68.5)	213 (70.1)	.67
	Not first child	189 (30.7)	98 (31.5)	91 (29.9)	
**Gestational age, mean (SD)**		9.00 (6.46)	8.61 (6.08)	9.39 (6.80)	.22
**Used fertility treatments to become pregnant**					
	Yes	35 (5.5)	17 (5.2)	18 (5.9)	.67
	No	599 (94.5)	313 (94.8)	286 (94.1)	
**ACE score**					
	Score greater than or equal to 4	113 (18.0)	64 (19.5)	49 (16.3)	.31
	Score less than 4	516 (82.0)	265 (80.5)	251 (83.7)	
**I am comfortable using a computer or laptop**					
	Very comfortable	591 (93.7)	311 (94.5)	280 (92.7)	.45
	Somewhat comfortable	36 (5.7)	17 (5.2)	19 (6.3)	
	Not very comfortable	4 (0.6)	1 (0.3)	3 (1.0)	
**I am comfortable using a computer tablet (eg, iPad)**					
	Very comfortable	530 (84.0)	280 (85.1)	250 (82.8)	.64
	Somewhat comfortable	89 (14.1)	44 (13.4)	45 (14.9)	
	Not very comfortable	12 (1.9)	5 (1.5)	7 (2.3)	
**I am comfortable using a smartphone**					
	Very comfortable	546 (86.5)	286 (86.9)	260 (86.1)	.32
	Somewhat comfortable	70 (11.1)	38 (11.6)	32 (10.6)	
	Not very comfortable	15 (2.4)	5 (1.5)	10 (3.3)	

^a^Some demographic data missing.

^b^Comparison of control and intervention groups: chi-square statistic used for variables with 3 or more categories; two-tailed *t* test used for variables with estimated means.

### Primary Objectives

#### Acceptability of E-Screening

More women in the e-screening group strongly or somewhat agreed that they would like to use or did like using a tablet for answering questions on emotional health (57.9%, 175/302 vs 37.2%, 121/325) and would or did prefer using a tablet to paper (46.0%, 139/302 vs 29.2%, 95/325), compared with women in the paper-based screening group. We observed, too, that fewer women who used the tablet answered *does not matter one way or the other* than women who completed paper-based screening on the items *I would or did like to use a tablet to answer questions about emotional health* (30.8%, 93/302 vs 53.5%, 174/325) and *I would prefer answering questions about emotional health on the tablet compared to a paper questionnaire* (40.4%, 122/302 vs 60.9%, 198/325).

#### Feasibility of E-Screening

##### Disclosure

Overall, women in both e-screening and paper-based screening groups indicated that they would be able to disclose their concerns about their mental health (Table 2). There was no significant difference between groups on the item *I was able to tell the truth on all the questions about emotional health*, with 94.1% (284/302) of women in the e-screening intervention group and 90.2% (293/325) in the paper-based control group somewhat or strongly agreeing they could tell the truth on all questions. In addition, few women in both groups indicated that they would find it difficult to answer how they felt with e-screening.

##### Features of Screening

Women in the e-screening group consistently reported the features of e-screening as superior to paper-based screening. For instance, significantly more women in the e-screening group (vs paper-based screening) reported they would like e-screening because it was private (64.6%, 195/302 vs 31.7%, 103/325) and they perceived the questions to be easier to understand (88.8%, 268/302 vs 87.7%, 285/325). Similarly, fewer women in the e-screening group reported they would find e-screening impersonal (3.6%, 11/302 participants vs 4.9%, 16/ 325 participants). Finally, significantly more women in the e-screening group reported they did not find screening too time-consuming, compared with women in the paper-based screening group (57.9%, 54/302 vs 40.9%, 40/325).

##### Comparison With Face-to-Face Screening

In both groups, less than 10% (24/302 and 30/325) of women acknowledged that it would be easier to have a nurse ask questions about emotional health compared with completing questions on a tablet. More than half the women in both groups preferred self-report e-screening compared with face-to-face screening, and this proportion was significantly greater in the e-screening group (58.9%, 178/302 participants vs 50.7%, 165/325).

**Table 2:** Feasibility and acceptability of e-screening.

Individual items of computer assessment evaluation scale		Full sample (N=627^a^), n (%)	Group 1: paper-based screening (n=325^a^), n (%)	Group 2: e-screening (n=302^a^), n (%)	*P* value^d^
**1. I would or did like to use a tablet to answer these** **questions about emotional health^b^**					
	Strongly disagree	42 (6.7)	23 (7.1)	19 (6.3)	<.001
	Somewhat disagree	22 (3.5)	7 (2.2)	15 (5.0)	
	Does not matter one way or the other	267 (42.6)	174 (53.5)	93 (30.8)	
	Somewhat agree	65 (10.4)	32 (9.8)	33 (10.9)	
	Strongly agree	231 (36.8)	89 (27.4)	142 (47.0)	
**2. I found the questions about emotional health easy to** **understand**					
	Strongly disagree	42 (6.7)	17 (5.2)	25 (8.3)	.03
	Somewhat disagree	13 (2.1)	10 (3.1)	3 (1.0)	
	Does not matter one way or the other	19 (3.0)	13 (4.0)	6 (2.0)	
	Somewhat agree	122 (19.5)	72 (22.2)	50 (16.6)	
	Strongly agree	431 (68.7)	213 (65.5)	218 (72.2)	
**3. I would not or did not like answering questions about** **emotional health on a tablet because it felt or would feel** **impersonal^b^**					
	Strongly disagree	306 (48.9)	139 (42.9)	167 (55.3)	.01
	Somewhat disagree	66 (10.5)	32 (9.9)	34 (11.3)	
	Does not matter one way or the other	227 (36.3)	137 (42.3)	90 (29.8)	
	Somewhat agree	17 (2.7)	10 (3.1)	7 (2.3)	
	Strongly agree	10 (1.6)	6 (1.8)	4 (1.3)	
**4. I would prefer answering questions about emotional** **health on a tablet compared to a paper questionnaire**					
	Strongly disagree	43 (6.9)	18 (5.5)	25 (8.3)	<.001
	Somewhat disagree	30 (4.8)	14 (4.3)	16 (5.3)	
	Does not matter one way or the other	320 (51.0)	198 (60.9)	122 (40.4)	
	Somewhat agree	63 (10.0)	27 (8.3)	36 (11.9)	
	Strongly agree	171 (27.3)	68 (20.9)	103 (34.1)	
**5. I would find it easier to answer the questions about** **emotional health on a tablet rather than having a nurse** **ask me questions**					
	Strongly disagree	24 (3.8)	12 (3.7)	12 (4.0)	.02
	Somewhat disagree	30 (4.8)	18 (5.5)	12 (4.0)	
	Does not matter one way or the other	230 (36.7)	130 (40.0)	100 (33.1)	
	Somewhat agree	173 (27.6)	96 (29.5)	77 (25.5)	
	Strongly agree	170 (27.1)	69 (21.2)	101 (33.4)	
**6. I did not like answering the questions about emotional** **health on the tablet or paper because it was hard to answer** **how I really felt^c^**					
	Strongly disagree	251 (40.0)	90 (27.7)	161 (53.3)	<.001
	Somewhat disagree	89 (14.2)	46 (14.2)	43 (14.2)	
	Does not matter one way or the other	218 (34.8)	152 (46.8)	66 (21.9)	
	Somewhat agree	60 (9.6)	30 (9.2)	30 (9.9)	
	Strongly agree	9 (1.4)	7 (2.2)	2 (0.7)	
**7. I would or did like answering questions about emotional** **health on a tablet because it would or did feel private^b^**					
	Strongly disagree	23 (3.7)	16 (4.9)	7 (2.3)	<.001
	Somewhat disagree	35 (5.6)	19 (5.8)	16 (5.3)	
	Does not matter one way or the other	271 (43.2)	187 (57.5)	84 (27.8)	
	Somewhat agree	155 (24.7)	66 (20.3)	89 (29.5)	
	Strongly agree	143 (22.8)	37 (11.4)	106 (35.1)	
**8. I did not like answering questions about emotional** **health on the tablet or on paper because the questions took** **too long for me to answer^c^**					
	Strongly disagree	213 (34.0)	93 (28.6)	120 (39.7)	<.001
	Somewhat disagree	95 (15.2)	40 (12.3)	55 (18.2)	
	Does not matter one way or the other	225 (35.9)	152 (46.8)	73 (24.2)	
	Somewhat agree	78 (12.4)	32 (9.8)	46 (15.2)	
	Strongly agree	16 (2.6)	8 (2.5)	8 (2.6)	
**9. I was able to tell the truth on all the questions about** **emotional health**					
	Strongly disagree	20 (3.2)	14 (4.3)	6 (2.0)	.25
	Somewhat disagree	6 (1.0)	5 (1.5)	1 (0.3)	
	Does not matter one way or the other	24 (3.8)	13 (4.0)	11 (3.6)	
	Somewhat agree	64 (10.2)	33 (10.2)	31 (10.3)	
	Strongly agree	513 (81.8)	260 (80.0)	253 (83.8)	

^a^Total sample missing data=9 (group 1: paper-based screening=6; group 2: e-screening=3).

^b^Women in the intervention group answered questions rating their experience of e-screening (eg, I *liked* answering questions about emotional health on a tablet because it felt private). Those in the control group answered questions to assess their views on e-screening (eg, I *would like* answering questions about emotional health on a tablet because it would feel private).

^c^Women in the intervention group answered questions regarding the experience of e-screening (eg, I did not like answering questions about emotional health on *the tablet* because the questions took too long for me to answer). Those in the control group answered questions regarding the experience of paper-based screening (eg, I did not like answering questions about emotional health on *paper* because the questions took too long for me to answer).

^d^Comparison of control and intervention groups: chi-square statistic used for all variables.

### Secondary Objectives

#### Factors Associated With Preference for E-Screening

Table 3 presents the results of the bivariate analysis of the association of each independent variable with the outcome, *I would or did like to use the tablet to answer these questions about emotional health*. Independent variables associated with the outcome at *P*<.20 were entered into the final multivariable logistic regression model, including mode of screening, maternal age, income, and education. In the final model, only mode of screening was significantly associated with the outcome, where the odds of preferring e-screening were 2.29 times greater for women in the e-screening group than women in the paper-based screening group. When we repeated this analysis using the outcome, *I would or did prefer answering questions about emotional health on the tablet compared to a paper questionnaire*, findings were similar to the odds of preferring e-screening estimated at 2.03 (95% CI 1.46-2.84).

**Table 3:** Factors associated with preference for e-screening based on the outcome, *I would or did like using a tablet to answer these questions about emotional health*.

Independent variables		Strongly or somewhat agree, n (%)	Does not matter, somewhat or strongly disagree^a^, n (%)	UOR^b^ (95% CI)	AOR^c^ (95% CI)
**Mode of report**					
	Electronic	176 (59.3)	127 (38.4)	2.34 (1.70-3.22)^d^	2.29 (1.66-3.17)
	Paper-based	121 (40.7)	204 (61.6)	1.00	1.00
**Recruitment site**					
	Community-based clinic or prenatal class	261 (89.1)	288 (88.9)	1.02 (0.62-1.69)	
	High-risk antenatal unit at tertiary care center	32 (10.9)	36 (11.1)	1.00	
**Age, years**					
	Less than 25	31 (10.5)	57 (17.2)	0.56 (0.35-0.90)^d^	0.68 (0.41-1.13)
	Greater than or equal to 25	265 (89.5)	274 (82.8)	1.00	1.00
**Income**					
	Less than $40,000	38 (12.8)	59 (17.8)	0.68 (0.44-1.06)^d^	0.77 (0.49-1.23)
	$40,000 or more	258 (87.2)	272 (82.2)	1.00	1.00
**Marital status**					
	Unpartnered	11 (3.7)	16 (4.8)	0.76 (0.35-1.67)	
	Partnered	285 (96.3)	315 (95.2)	1.00	
**Diagnosis of depression, anxiety, or any other kind of** **emotional concern**					
	Yes	74 (24.9)	88 (26.6)	0.92 (0.64-1.31)	
	No	223 (75.1)	243 (73.4)	1.00	
**Ever treated for depression, anxiety, or any other** **kind of emotional concern**					
	Yes	81 (27.3)	97 (29.3)	0.91 (0.64-1.28)	
	No	216 (72.7)	234 (70.7)	1.00	
**Ethnicity**					
	Not white	78 (26.4)	89 (26.9)	0.97 (0.68-1.39)	
	White	218 (73.6)	242 (73.1)	1.00	
**Born in Canada**					
	No	61 (20.6)	57 (17.2)	1.25 (0.84-1.86)	
	Yes	235 (79.4)	274 (82.8)	1.00	
**Pregnant before**					
	First child	197 (67.7)	228 (70.8)	0.86 (0.61-1.22)	
	Not first child	94 (32.3)	94 (29.2)	1.00	
**Education**					
	High school or less	37 (12.5)	63 (19.0)	0.61 (0.39-0.94)^d^	0.73 (0.45-1.18)
	Some postsecondary or more	259 (87.5)	268 (81.0)	1.00	1.00
**Used fertility treatments to become pregnant**					
	Yes	18 (6.1)	17 (5.1)	1.19 (0.60-2.36)	
	No	279 (93.9)	314 (94.9)	1.00	
**ACE score**					
	Score greater than or equal to 4	49 (16.7)	63 (19.0)	0.85 (0.56-1.28)	
	Score less than 4	245 (83.3)	268 (81.0)	1.00	

^a^The categories *does not matter* and *somewhat or strongly disagree* were combined to address low cell sizes in the *somewhat or strongly disagree* category for some variables.

^b^UOR: unadjusted odds ratio.

^c^AOR: adjusted odds ratio.

^d^Independent variables associated with the outcome at *P*<.20 were entered into the final multivariable logistic regression model, including mode of screening, maternal age, income, and education.

#### Disclosure

Table 4 presents the results of the bivariate analysis of the association of each independent variable with the outcome, *I was able to tell the truth on all the questions about emotional health*. Independent variables associated with the outcome at *P*<.20 were entered into the final multivariable logistic regression model, including mode of screening and education. In the final model, no variables were significantly associated with a woman’s ability to be honest during mental health screening.

**Table 4:** Factors associated with disclosure of mental health concerns during screening based on the outcome, *I was able to tell the truth on all questions about emotional health*.

Independent variables		Strongly or somewhat agree, n (%)	Does not matter, somewhat or strongly disagree^a^, n (%)	UOR^b^ (95% CI)	AOR^c^ (95% CI)
**Mode of report**					
	Electronic	285 (49.3)	18 (36.0)	1.73 (0.95-3.15)^d^	1.69 (0.92-3.08)
	Paper-based	293 (50.7)	32 (64.0)	1.00	1.00
**Recruitment site**					
	Community-based clinic or prenatal class	507 (89.1)	42 (87.5)	1.17 (0.48-2.86)	
	High-risk antenatal unit at tertiary care center	62 (10.9)	6 (12.5)	1.00	
**Age, years**					
	Less than 25	80 (13.9)	8 (16.0)	0.85 (0.38-1.87)	
	Greater than or equal to 25	497 (86.1)	42 (84.0)	1.00	
**Income in Can $**					
	Less than $40,000	90 (15.6)	7 (14.0)	1.14 (0.50-2.60)	
	$40,000 or more	487 (84.4)	43 (86.0)	1.00	
**Marital status**					
	Unpartnered	25 (4.3)	2 (4.0)	1.09 (0.25-4.73)	
	Partnered	552 (95.7)	48 (96.0)	1.00	
**Diagnosis of depression, anxiety, or any other kind of** **emotional concern**					
	Yes	150 (26.0)	12 (24.0)	1.11 (0.57-2.18)	
	No	428 (74.0)	38 (76.0)	1.00	
**Ever treated for depression, anxiety, or any other** **kind of emotional concern**					
	Yes	164 (28.4)	14 (28.0)	1.02 (0.54-1.94)	
	No	414 (71.6)	36 (72.0)	1.00	
**Ethnicity**					
	Not white	151 (26.2)	16 (32.0)	0.75 (0.40-1.40)	
	White	426 (73.8)	34 (68.0)	1.00	
**Born in Canada**					
	No	110 (19.1)	8 (16.0)	1.24 (0.57-2.71)	
	Yes	467 (80.9)	42 (84.0)	1.00	
**Pregnant before**					
	First child	394 (70.0)	31 (62.0)	1.43 (0.79-2.60)	
	Not first child	169 (30.0)	19 (38.0)	1.00	
**Education**					
	High school or less	87 (15.1)	13 (26.0)	0.51 (0.26-0.99)^d^	0.52 (0.27-1.02)
	Some postsecondary or more	490 (84.9)	37 (74.0)	1.00	1.00
**Used fertility treatments to become pregnant**					
	Yes	32 (5.5)	3 (6.0)	0.92 (0.27-3.11)	
	No	546 (94.5)	47 (94.0)	1.00	
**ACE score**					
	Score greater than or equal to 4	106 (18.4)	6 (12.0)	1.66 (0.69-3.99)	
	Score less than 4	469 (81.6)	44 (88.0)	1.00	

^a^The categories *does not matter* and *somewhat or strongly disagree* were combined to address low cell sizes in the *somewhat or strongly disagree* category for some variables.

^b^UOR: unadjusted odds ratio.

^c^AOR: adjusted odds ratio.

^d^Independent variables associated with the outcome at *P*<.20 were entered into the final multivariable logistic regression model.

## Discussion

### Principal Findings

The findings of this study suggest that e-screening is a feasible approach to mental health screening. Overall, women in both e-screening and paper-based screening groups indicated that they would be able to disclose their concerns about their mental health, with women in the e-screening group consistently reporting the features of e-screening as superior to paper-based screening. In the multivariable analyses, we found that women in the e-screening group were more likely to prefer e-screening compared with women in the paper-based screening group. However, none of the independent variables, including demographics, mental health history, mode of screening, or obstetric history, were significantly associated with women’s ability to be honest during screening.

### Primary Outcomes

#### Acceptability of E-Screening

More women in the e-screening group favored the use of the tablet for mental health screening and indicated a preference for e-screening over paper than women in the paper-based screening group. This is consistent with our previous studies, in which 86% of pregnant women surveyed indicated that they would be very or somewhat comfortable answering questions on a computer or iPad [61].

It is also interesting that more women who were randomized to the e-screening group responded with a more defined preference for e-screening (eg, higher proportion agreed, lower proportion reported that it did not matter one way or the other), compared with women in the paper-based screening group. This finding may relate to the benefit that those in the e-screening group experienced, compared with women in the paper-based screening group who anticipated their responses to e-screening. This finding is important from an implementation perspective when considering women’s initial responses to e-screening. While women who have never completed e-screening may initially be ambivalent, our evidence suggests that the actual experience may prove to be an easier, more comfortable, and more private experience than anticipated. This information (eg, once women experience e-screening, they tend to “like it”) may also be valuable to share with women who express ambivalence about e-screening.

#### Feasibility of E-Screening

##### Disclosure

The vast majority of women in both groups reported that they were able to tell the truth on all questions about emotional health. We were interested in this aspect of screening because previous qualitative studies of paper-based and face-to-face screening found that some pregnant [62] and postpartum [62,63] women purposefully limited their disclosure of current or previous mental health concerns during the screening process. We wanted to quantify the magnitude of this issue, given that the ability of screening tools to accurately identify women with potential mental health problems depends on women being honest in the first place about their status. Our trial’s finding is consistent with a previous cross-sectional study we conducted (N=460) in which 79% of pregnant women indicated they could be completely honest if their prenatal care provider asked them about their mental health [20]. Multivariable analysis in that study also showed that the level of honesty (completely vs somewhat or not at all) women anticipated they would have during screening did not vary depending on whether questions were asked on paper or tablet or computer [20]. We found no other studies that assessed disclosure of mental health issues in pregnancy during e-screening. Our findings align with Renker and Tonkin’s conclusions that postpartum women were able to disclose interpersonal violence during a computer-administered interpersonal screening assessment [47].

##### Features of Screening

All of the features of e-screening that we assessed were viewed more favorably by the e-screening group, suggesting that these women felt that e-screening was a superior approach to screening than paper-based screening. Significantly more women in the e-screening group perceived that screening was not too time-consuming and that e-screening had the benefits of being more private than paper-based screening. These findings are similar to one study of computerized violence screening where postpartum women reported that the features of computer screening made it easier for them to answer questions about violence, compared with face-to-face or written approaches [47]. Fewer women in the e-screening group also indicated that they found e-screening impersonal, which is positive given that, anecdotally, clinicians have expressed concerns that women might find e-screening too impersonal to divulge clinically important information.

We found no other studies with which to compare our findings regarding pregnant women’s (or others’) perceptions of the features of mental health e-screening. However, women’s views on these features of e-screening do not appear to influence its clinical benefit. Although women in the e-screening group found e-screening to have more optimal features than a paper-based approach, both groups indicated they could disclose their mental health concerns, regardless of mode of screening. From another perspective, however, women’s perceptions of the benefits of e-screening may impact uptake of screening and therefore screening rates. For instance, if women perceive e-screening to be a personal, private experience of easy-to-manage questions, they may be more willing to consent to screening as part of routine prenatal care. Certainly, issues related to the process of screening have been previously identified as deterrents to screening. For example, in a qualitative study examining the acceptability of face-to-face postnatal depression screening, some women described the lack of privacy during screening as inhibiting and inappropriate, while others described the face-to-face approach as intrusive. In addition, e-screening may eliminate some of the barriers to screening engagement that women have identified regarding poor attitudes and negative judgment of the health care professional conducting the screening [62,63].

##### Comparison With Face-to-Face Screening

In this study, less than 10.0% (24/302 or 30/325) of women in each group indicated that it was easier to answer questions on emotional health when asked face-to-face, in this case by a nurse (compared with on a tablet), more than half in each group stated a preference for e-screening over nurse-led screening, and less than 5.0% (11/302 or 16/324) reported that they would not like e-screening because it would feel impersonal. Taken together, these findings suggest that women prefer self-report methods over face-to-face approaches for mental health screening. A previous cross-sectional study that we conducted in a sample of 460 pregnant women recruited across the province of Alberta (Canada) showed similar findings in that while more women were comfortable with a variety of self-report options (paper, e-screening, completion at home and sending to clinic, completion in clinic waiting room), the mode of screening that garnered the lowest level of comfort was being called at home by a nurse [61]. Findings of this trial are also consistent with a cross-sectional study conducted by Renker and Tonkin, which reported that, in general, postpartum women “overwhelmingly supported” computer-administered interpersonal screening assessment over face-to-face and written approaches [47].

The qualitative study by Rollans et al [62] reported that, during the process of mental health screening by a midwife, some pregnant women found the midwives’ responses when they disclosed sensitive information distressing. At times, it was the response of the midwife or nurse to a woman’s disclosure that caused her the most distress. This study also found that women’s perceptions of the midwives’ approach influenced their level of comfort with screening, especially when they felt like they were “being watched.” In light of the fact that women did not view e-screening as impersonal, and perceived a potential risk with face-to-face screening, e-screening may offer a more acceptable approach to screening.

### Secondary Outcomes

#### Factors Associated With Women’s Preferences for E-Screening

The finding that none of the characteristics we assessed, including demographics, mental health history, and obstetric history, were significantly related to women’s preferences for e-screening suggests that most women would accept being screened using either mode. The only factor significantly associated with women’s preference for e-screening was being in the e-screening group. This result suggests that women who actually experienced e-screening were more likely to prefer it. This should offer some reassurance in e-screening implementation in the clinical setting in that it indicates that once women use e-screening they tend to favor it.

#### Factors Associated With Women’s Disclosure During Screening

No factors we assessed were significantly associated with women’s ability to be honest during screening, including mode of screening (e-screening vs paper-based screening). As such, we did not identify any subgroups of women who might be less apt to disclose their mental health concerns during screening. This finding suggests that women across all ages, income, and education, those with a mental health history, and those with obstetric complications would be comfortable with being honest during mental health screening. The lack of association between mode of screening and disclosure indicates that both paper-based and e-screening approaches to screening facilitate disclosure. These findings are similar to those of our cross-sectional study, in which we also found that neither e-screening nor paper-based screening were related to disclosure [20]. This is positive, in that it suggests that clinical settings can select whichever mode best suits their service delivery model without hampering disclosure.

### Limitations

In order to limit participant burden, we evaluated the e-screening and paper-based versions of the ALPHA and the EPDS. Although these 2 screening tools are widely used in perinatal clinical settings, evaluation of other tools is warranted (eg, Generalized Anxiety Disorder–7 scale, Whooley Questions, Antenatal Risk Questionnaire). Overall, women in our study tended to be well-educated, partnered, and affluent, which may limit the generalizability of the findings. However, in our multivariable analyses, these factors were not important influences in women’s perceptions of the acceptability and feasibility of mode of mental health screening. We also excluded women who did not speak English as the first step to trial e-screening. Indeed, one of the most important applications of e-screening may be the feature of having immigrant women answer questions in their preferred language and devising a computer-based algorithm to assess the scale score and interpret it automatically for the English-speaking provider. Future studies should evaluate this application of e-screening.

### Conclusions

The final participation rate for the trial was 94.2% (636/675), suggesting that most women were eager to participate in a trial of screening for mental health concerns. This trial’s findings support the feasibility and acceptability of e-screening among pregnant women, suggesting that it is a viable service delivery option for mental health screening in busy, primary care settings. As an implementation consideration, clinic and hospital staff would require minimal training to support women in accessing the Web-based screening link on the tablet computer. Future studies should evaluate the effectiveness of e-screening on clinical outcomes, including follow-up assessment, linkage to services, and reduction of risk of mental health disorders.

## Appendix

Multimedia Appendix 1 Screenshot of the Edinburgh Postnatal Depression Scale.

## Abbreviations

ALPHA: Antenatal Psychosocial Health Assessment
CIHR: Canadian Institutes of Health Research
EPDS: Edinburgh Postnatal Depression Scale
MINI: Mini-International Neuropsychiatric Interview
WCHRI: Women and Children’s Health Research Institute
