# Supplementary material for: Pregnant Women’s Views on the Feasibility and Acceptability of Web-Based Mental Health E-Screening Versus Paper-Based Screening: A Randomized Controlled Trial
Source: J Med Internet Res. 2017 Apr 7;19(4):e88. doi: 10.2196/jmir.6866 (PMC5400885; doi:10.2196/jmir.6866)
Supplement: Multimedia Appendix 1 [file jmir_v19i4e88_app1.pdf]

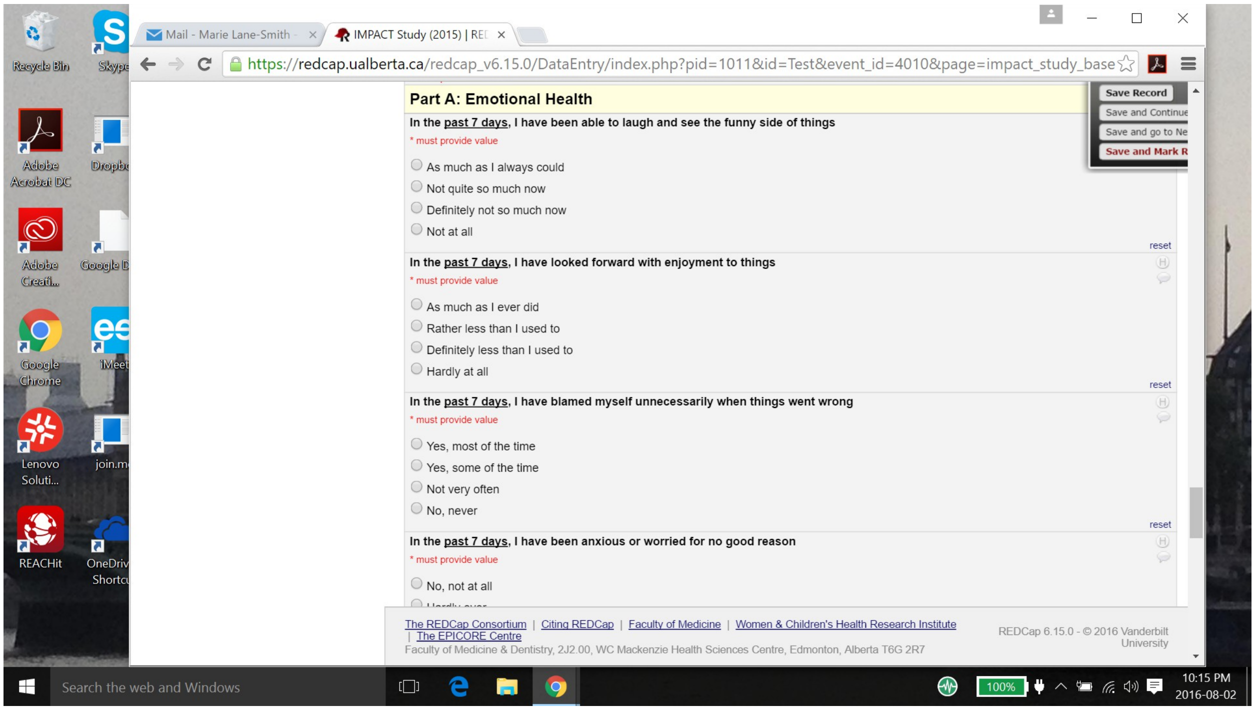

https://redcap.ualberta.ca/redcap_v6.15.0/DataEntry/index.php?pid=1011&id=Test&event_id=4010&page=impact_study_base

Part A: Emotional Health

In the past 7 days, I have been able to laugh and see the funny side of things
* must provide value
- As much as I always could
- Not quite so much now
- Definitely not so much now
- Not at all

In the past 7 days, I have looked forward with enjoyment to things
* must provide value
- As much as I ever did
- Rather less than I used to
- Definitely less than I used to
- Hardly at all

In the past 7 days, I have blamed myself unnecessarily when things went wrong
* must provide value
- Yes, most of the time
- Yes, some of the time
- Not very often
- No, never

In the past 7 days, I have been anxious or worried for no good reason
* must provide value
- No, not at all

The REDCap Consortium | Citing REDCap | Faculty of Medicine | Women & Children's Health Research Institute | The EPICORE Centre
Faculty of Medicine & Dentistry, 2J2.00, WC Mackenzie Health Sciences Centre, Edmonton, Alberta T6G 2R7
REDCap 6.15.0 - © 2016 Vanderbilt University

10:15 PM 2016-08-02

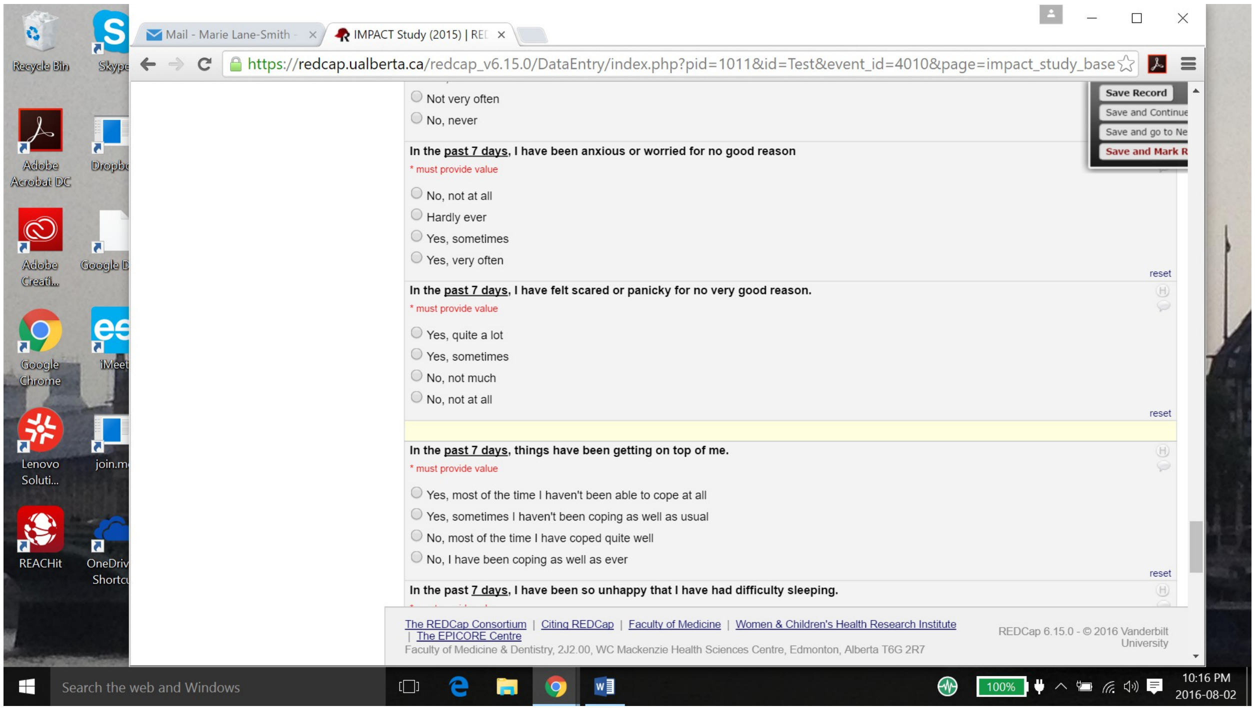

https://redcap.ualberta.ca/redcap_v6.15.0/DataEntry/index.php?pid=1011&id=Test&event_id=4010&page=impact_study_base

- Not very often
- No, never

In the past 7 days, I have been anxious or worried for no good reason
* must provide value
- No, not at all
- Hardly ever
- Yes, sometimes
- Yes, very often

In the past 7 days, I have felt scared or panicky for no very good reason.
* must provide value
- Yes, quite a lot
- Yes, sometimes
- No, not much
- No, not at all

In the past 7 days, things have been getting on top of me.
* must provide value
- Yes, most of the time I haven't been able to cope at all
- Yes, sometimes I haven't been coping as well as usual
- No, most of the time I have coped quite well
- No, I have been coping as well as ever

In the past 7 days, I have been so unhappy that I have had difficulty sleeping.

The REDCap Consortium | Citing REDCap | Faculty of Medicine | Women & Children's Health Research Institute | The EPICORE Centre
Faculty of Medicine & Dentistry, 2J2.00, WC Mackenzie Health Sciences Centre, Edmonton, Alberta T6G 2R7
REDCap 6.15.0 - © 2016 Vanderbilt University

10:16 PM 2016-08-02

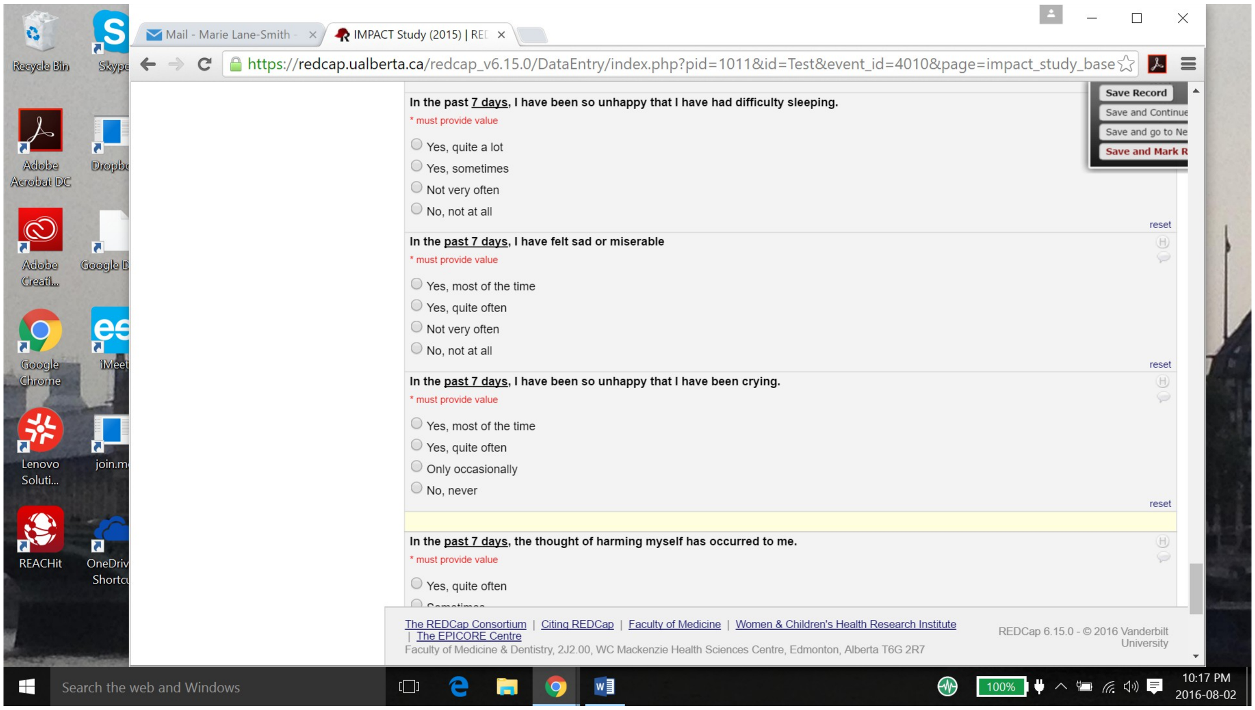

https://redcap.ualberta.ca/redcap_v6.15.0/DataEntry/index.php?pid=1011&id=Test&event_id=4010&page=impact_study_base

Save Record
Save and Continue
Save and go to Ne
Save and Mark R

In the past 7 days, I have been so unhappy that I have had difficulty sleeping.
* must provide value

- Yes, quite a lot
- Yes, sometimes
- Not very often
- No, not at all

reset

In the past 7 days, I have felt sad or miserable
* must provide value

- Yes, most of the time
- Yes, quite often
- Not very often
- No, not at all

reset

In the past 7 days, I have been so unhappy that I have been crying.
* must provide value

- Yes, most of the time
- Yes, quite often
- Only occasionally
- No, never

reset

In the past 7 days, the thought of harming myself has occurred to me.
* must provide value

- Yes, quite often

The REDCap Consortium | Citing REDCap | Faculty of Medicine | Women & Children's Health Research Institute | The EPICORE Centre
Faculty of Medicine & Dentistry, 2J2.00, WC Mackenzie Health Sciences Centre, Edmonton, Alberta T6G 2R7
REDCap 6.15.0 - © 2016 Vanderbilt University

10:17 PM 2016-08-02

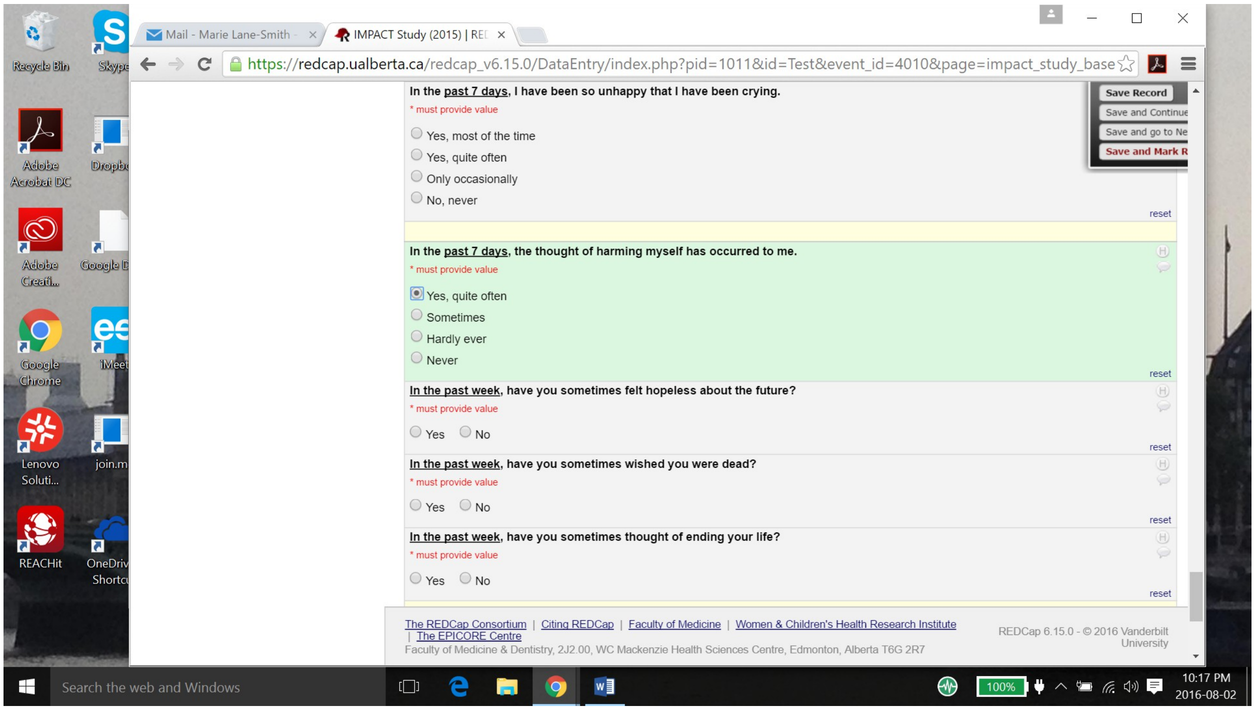

https://redcap.ualberta.ca/redcap_v6.15.0/DataEntry/index.php?pid=1011&id=Test&event_id=4010&page=impact_study_base

Save Record
Save and Continue
Save and go to Ne
Save and Mark R

In the past 7 days, I have been so unhappy that I have been crying.
* must provide value

- Yes, most of the time
- Yes, quite often
- Only occasionally
- No, never

reset

In the past 7 days, the thought of harming myself has occurred to me.
* must provide value

- Yes, quite often
- Sometimes
- Hardly ever
- Never

reset

In the past week, have you sometimes felt hopeless about the future?
* must provide value

Yes No

reset

In the past week, have you sometimes wished you were dead?
* must provide value

Yes No

reset

In the past week, have you sometimes thought of ending your life?
* must provide value

Yes No

reset

The REDCap Consortium | Citing REDCap | Faculty of Medicine | Women & Children's Health Research Institute | The EPICORE Centre
Faculty of Medicine & Dentistry, 2J2.00, WC Mackenzie Health Sciences Centre, Edmonton, Alberta T6G 2R7
REDCap 6.15.0 - © 2016 Vanderbilt University

10:17 PM 2016-08-02
